# Bronchial Dieulafoy’s disease: a retrospective analysis of 73 cases

**DOI:** 10.1186/s12890-019-0863-1

**Published:** 2019-06-06

**Authors:** Xin Qian, Qiong Du, Na Wei, Meifang Wang, Hansheng Wang, Yijun Tang

**Affiliations:** 1 Department of Pulmonary and Critical Care Medicine, Taihe Hospital, Hubei University of Medicine, 32 Renmin South Road, Shiyan, 442000 Hubei Province China; 2 Respiratory Endoscopy Center, Taihe Hospital, Hubei University of Medicine, 32 Renmin South Road, Shiyan, 442000 Hubei Province China

**Keywords:** Bronchial Dieulafoy’s disease, Bronchoscopy, Vascular angiography, Bronchial artery embolization, Surgery

## Abstract

**Background:**

Bronchial Dieulafoy’s disease (BDD) is a rare disease that is known to be a cause of hemorrhage. The characteristics of this disease are still unknown. The present study describes the disorder based on a review of the world’s literature, emphasizing the diagnostic and therapeutic views.

**Methods:**

A comprehensive research of BDD of the PubMed, Google Scholar, and Web of Science databases was performed. The following data were collected: patient characteristics; chest imaging, bronchoscopy, vascular angiography, and histopathologic examination findings; and treatment rendered.

**Results:**

73 cases of BDD have been reported from 1995 to 2019. Most of the cases occurred in Asia (52.1%), followed by Europe (31.5%). Chest imaging findings were non-specific. The main bronchoscopy finding was a nodular or protruding lesion (60.9%). 19 patients underwent bronchoscopic biopsies, 17 had bleeding, and 6 died. Four patients were successfully shown to have vascular malformations under mucosal protrusion by endobronchial ultrasound scan (EBUS). Vascular angiography mainly showed tortuous, dilated bronchial arteries. Vascular angiography mainly showed tortuous, dilated bronchial arteries. The arterial supply was mainly provided by bronchial arteries (48 cases) and the pulmonary circulation (4 cases). The lesions were mainly located in the right bronchus (53 cases). Selective bronchial artery embolization (BAE) was attempted in 38 patients and 20 patients underwent lobectomies. Emergency resection was performed in 15 patients, all of whom survived and had no recurrent hemoptysis.

**Conclusions:**

Massive hemoptysis was the common manifestation of BDD. Vascular angiography and EBUS is a very useful examination before biopsy. BAE may be used in stable patients, or patients who cannot tolerate surgery, while surgical resection should be considered in patients who are unstable, patients with uncontrolled hemoptysis, or following BAE failure.

**Electronic supplementary material:**

The online version of this article (10.1186/s12890-019-0863-1) contains supplementary material, which is available to authorized users.

## Background

In 1898, Georges Dieulafoy first reported a superficial gastric ulcer for vascular malformations, and described the presence of a vascular anomaly characterized by the existence of dilated, tortuous arteries in the submucosa [1]. Dieulafoy’s disease is extremely rare. The usual affected site of Dieulafoy’s disease is the digestive tract, including the esophagus, duodenum, jejunum, gallbladder, colon, and rectum [2]. Sweerts et al. first reported bronchial Dieulafoy disease (BDD) in 1995 [3].

Over the past decade, BDD has been increasingly recognized as a cause of pulmonary hemorrhage. The etiology of this disease is still unknown. Current theories regarding the etiology of BDD include congenital vascular malformations or chronic bronchial injury secondary to previous pulmonary infections [3]; however, age and tobacco use have an influence on BDD [4, 5]. Hemoptysis is the most common presentation [6]. The usual bronchoscopic appearance is that of a small sessile lesion covered with bronchial mucosa and a white cap [7, 8].

There has been no consensus for treatment of BDD. Selective bronchial artery embolization (BAE) has been proposed as a method for stopping the bleeding, but BAE may fail and bleeding may recur [9]. Surgical resection should be considered in patients who are unstable, patients with uncontrolled hemoptysis, or following BAE failure [6].

To date, few case reports and case series involving BDD have been published, hence the aim of the current study was to analyze the clinical presentation, diagnosis, and treatment characteristics of BDD cases worldwide.

## Methods

A comprehensive search of the PubMed, Google Scholar and Web of Science databases was conducted to identity relevant studies published before February 12, 2019 using the following terms: “trachea OR airways OR bronchus OR lung OR bronchial tree” AND “bleeding OR massive hemorrhage OR Dieulafoy’s disease”. In addition, the references from the retrieved articles that matched our inclusion criteria were manually searched, and the results were restricted to articles available in English abstract. Since the first report of BDD in 1995, there have been 73 reported cases domestically and internationally. 76 cases were reported in 45 publications, of which three cases were repeats, and two original articles reported 11 cases. Therefore, there have been 73 unique cases after excluding the repeat cases.

## Results

### Gender distribution and age at diagnosis

**Fig. 1:**
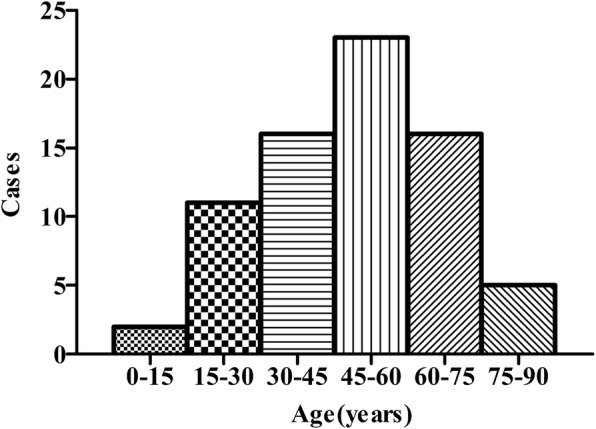
Distribution by age of 73 cases of Dieulafoy’s disease

73 cases of BDD were collected from 1995 to 2019, including 48 males and 25 females. The male-to-female ratio was approximately 2:1. The minimum and maximum ages at the time of diagnosis were 8 months and 85 years, respectively, with an average of 47.2 years. The majority of patients (31.5%) were between 45 years and 60 years of age, followed by 60–75 years (21.9%), and 30–45 years (21.9%). The disorder affected people at every age, but mainly at middle-aged adults, as shown in (Fig. 1).

### Continent and country of origin

**Fig. 2:**
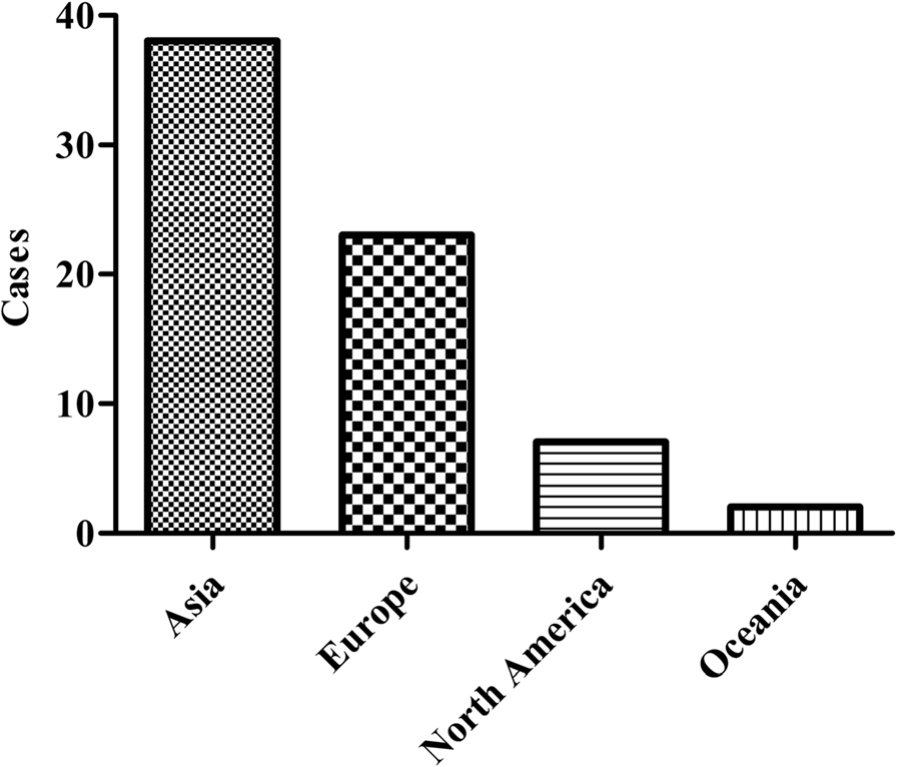
Dieulafoy’s disease in the international literature: subdivision by continent

Most of the cases were reported from Asia (38 patients) [6–24], followed by Europe (23 patients) [3, 4, 25–37], North America (seven patients) [38–44], Oceania (two patients) [5, 45], and the country of origin was not reported for three patients (Fig. 2) [46]. 15 countries contributed to the number of cases with 1–29 patients (Table 1). Of these countries, four had ≥ 5 patients [China, 29 patients; France, eight patients; UK, seven patients; and USA, six patients] (Table 1).

**Table 1:** Cases of Dieulafoy’s disease from 15 countries

Country	No. of cases	Country	No. of cases
UK	7	France	8
Switzerland	2	Netherlands	1
Germany	1	Italy	3
Spain	1	Turkey	3
China	29	India	1
Iran	4	Mexico	1
USA	6	Australia	2
Japen	1	Not Available	3

### Relationships between a history of smoking, other respiratory causes, and hemoptysis

35 of the 73 cases were smokers, 23 patients were never-smokers, and 15 pcs out of 73 (20.5%) were not known about their smoking history. 21 patients had a history of respiratory disorders, including tuberculosis, bronchiectasis, bronchitis, asthma, and COPD. 42 patients had a negative history for respiratory disorders, and the anamnesis for respiratory diseases not known for 10 out of 73 (13.7%) (Additional file 1: Table S1). We are therefore of the opinion that the etiology and pathogenesis of BDD may be related to chronic inflammation of the airways, respiratory tract injury, and long-term smoking.

### Reason for admission

70 of 73 patients had specific indications for hospital admission. Hemoptysis was the most common indication for hospital admission [60 patients (85.7%)], of which 37 patients had previous bleeding episode, 16 cases had not, and 7 cases did not report former bleeding history. Ten patients were admitted for other respiratory disorders, including eight patients without a history of hemoptysis (Table 2).

**Table 2:** Reasons for admission (*n* = 70)

Reasons	No. of cases	Proportion (%)
Hemoptysis	60	85.7
with previous bleeding	37	52.9
without previous bleeding	16	22.9
Pneumonia	4	5.7
Dyspnea	1	1.4
Bronchiectasis and bronchitis	2	2.9
Cough	2	2.9
Chest pain	1	1.4

### Imaging of Dieulafoy’s disease

An exhaustive diagnostic evaluation was performed with chest X-rays, computed tomography (CT) scans, bronchoscopies, biopsies, and angiographies. Of note, chest X-rays and CT scans did not establish the diagnosis of Dieulafoy’s disease. Although X-rays were not reported in 45 pcs out of 73 (61.6%), 28 patients were characterized; 16 patients had normal findings, ten had opacities, one had a mass lesion, and one had peribronchial lymphadenopathy. 60 patients had CT scans, the hallmarks of which were ground glass opacities (24 cases), followed by normal findings (9 cases), flaky shadows (6 cases), spotted shadows (2 cases), nodular lesions (2 cases), mass lesions (2 cases), abnormal bronchi (1 case), hypertrophied artery supplying (1 cases), other atypical findings (13 cases), and 13 cases out of 73 (17.8%) were not known (Additional file 1: Table S1). Thus, chest X-rays and CT scans are not first-line modalities by which to diagnose Dieulafoy’s disease due to lack of specificity and sensitivity.

64 patients underwent bronchoscopies. The main findings included the following three categories, as shown in Table 3: (1) a nodular or protruding lesion [39 patients (60.9%)]; (2) only clot or blood [15 patients (23.4%)]; and (3) bleeding points [5 patients (7.8%)].

**Table 3:** Bronchoscopy appearances of Dieulafoy’s disease (*n* = 64)

Bronchoscopy appearances	No. of cases	Proportion (%)
Only clot or blood	15	23.4
Nodular or protruding lesion	39	60.9
Bleeding point	5	7.8
Normal	2	3.1
Tortuous vessel	2	3.1
Only white cap	1	1.6

Additionally, 19 pcs out of 57 underwent bronchoscopic biopsies, 17 of whom had bleeding after the biopsy, including six deaths due to massive hemorrhage. The data on “bleeding on biopsy” is not known in 16 pcs out of 73 (21.9%). Only 5 patients biopsies specimens showed part of the wall of a small arterial vessel, however, they are mostly normal and nondiagnostic. The biopsies were mostly not ideal due to contaminated by bleeding; thus, performing biopsies would not be useful in this context, but carry the risk of triggering sudden massive hemoptysis.

Thirty-three patients underwent angiographies, 5 patients did not take, and 35 pcs out of 73 (47.9%) were not reported. Most of the angiographies revealed a tortuous, dilated bronchial artery, but two cases were normal. Thus, angiography has a role in the diagnosis of BDD, but cannot serve as the gold standard for diagnosis. Moreover, the source of abnormal vessels was reported in 53 cases. Four cases showed the source of tortuous, dilated vessels from the pulmonary artery, 48 cases were from the bronchial artery, and 20 pcs out of 73 (27.4%) did not report the source of the bleeding.

### Lung segment localization

The abnormal lesions were most often located in the right bronchus. Because four patients had disorders in the left and right lungs concurrently, we used the clinical manifestation rate (CMR) to show the probability of one specific localization of the lesion appearing in 73 patients, where CMR = (cases of one specific localization/72) *100%. The most common lobe localization in BDD was located in the right bronchus (53 cases; CMR = 73.6%), which included 22 cases in the right lower lobe (CMR = 30.6%), 16 cases in the right middle lobe (CMR = 22.2%), 15 cases in the right upper lobe (CMR = 20.8%), three cases in the right main bronchus (CMR =4.2%), and three cases in the right intermediate bronchus (CMR = 4.2%). The second most frequent lobe localization was in the left bronchus (22 cases; CMR = 30.6%), which included ten cases in the left lower lobe (CMR = 13.9%), nine cases in the left upper lobe (CMR =12.5%), and two cases in the left main bronchus (CMR = 2.8%). An extremely rare case was shown in the near carina (Table 4). Thus, the possibility of BDD should be suspected in patients who present with unexplained hemoptysis and in whom the lesion is in the right bronchus; unnecessary and hazardous procedures should be avoided in such patients. Furthermore, if such lesions occur in rare places, such as the carina and intermediate bronchus, we also should proceed with caution.

**Table 4:** Lung segment localization of Dieulafoy’s disease (*n* = 72)

Lung segment Localization	No. of cases	CMR (%)
Left bronchus	22	30.6
Left lower lobe	10	13.9
Left upper lobe	9	12.5
Left main bronchus	2	2.8
Right bronchus	53	73.6
Right lower lobe	22	30.6
Right middle lobe	16	22.2
Right upper lobe	15	20.8
Right main bronchus	3	4.2
Right intermediate bronchus	3	4.2
Near carina	1	1.4
Both lungs	4	5.6

CMR (clinical manifestation rate) = (cases of one specific localization/48) *100%

### Treatment of Dieulafoy’s disease

Of the 73 cases of BDD, there were 64 in whom a specific treatment was reported in the following six categories: (1) conservative treatment (six cases); (2) interventional bronchoscopy (five cases); (3) only BAE (18 cases); (4) only lobectomy (15 cases); (5) BAE+ lobectomy (20 cases); and (6) placement of a dumon silicone stent (one cases). Only three patients with massive hemoptysis received conservative treatment, had complete remission of symptoms, and another three patients without hemoptysis also received conservative treatment. Flexible bronchoscopic argon plasma coagulation was performed to successfully manage two cases. A Nd: YAP laser was used in one patient without further bleeding episodes at 6 months follow-up. Contact electrocautery temporized a bleeding lesion in one patient, but re-bleeding occurred shortly thereafter necessitating emergent lobectomy with definitive control. The cryotherapy was taken in only one case, but failed, and a dumon silicone stent was placed to this patient laterly. First-line selective therapeutic BAE was attempted in 38 patients, but 20 underwent further lobectomy as BAE was not successful or there were subsequent bleeding recurrences after BAE. Emergency resection was performed in 15 patients, all of them survived and had no relapse hemoptysis. Selective therapeutic BAE is often performed as first-line treatment to control hemoptysis, which is effective for partial patients; however, some patients may have recurrent hemoptysis and need to undergo surgery.

### Histological examination

33 cases performed histological examinations which included postoperative histopathological and autopsy histological examinations, 19 cases did not take histological examinations, and 21 cases out of 73 cases (28.8%) were not known. The results of biopsy specimens most often showed normal bronchial mucosa with conserved structures. Only five biopsies showed part of a wall of a small arterial vessel. With respect to histopathological examinations, an abnormal vessel was macroscopically visible within the bronchial wall in 27 cases, including the abnormal vessel clearly opening into the lumen in nine cases. Although postoperative histopathological examination is the gold standard for the diagnosis of BDD, the need for pathological diagnosis remains controversial.

## Discussion

Bronchial Dieulafoy’s disease (BDD) is a rare vascular anomaly consisting of a dysplastic artery in the submucosa. The numerous published reports have dealt only with a small number of cases; the actual incidence may be higher because the condition is underappreciated and underdiagnosed [25]. The natural history of this disease, diagnostic methods, and the preferred treatment are not well-known; however, this disease has been increasingly recognized as a cause of pulmonary hemorrhage.

The etiology and pathogenesis of BDD are uncertain, and whether or not BDD is congenital or acquired is the subject of debate. The male-to-female ratio is approximately 2:1 and middle-aged adults are most frequently affected, indicating that gender or age is closely associated with the disease process. A large proportion of patients are smokers (35/58) and had existing respiratory diseases (21/63), suggesting that chronic airway inflammation, related to smoking or chronic infection, have an influence on this disease [31]. Furthermore, this disease most often occurs in Asia [38 patients (52.1%)] and Europe [23 patients (31.5%)] of the seven continents, thus may have a correlation with the tobacco use rate. It has been suggested that the possibility of an acquired vascular malformation is the result of chronic bronchial injury, although the direct role of chronic inflammation in the evolution of the vascular pathology is ambiguous.

However, the lowest age at the time-of-diagnosis of BDD was 8 months (i.e., no history of smoking or respiratory disorder). And, a number of the reported cases have had no evidence of a previous pulmonary infection or associated malformations. In the absence of an obvious cause, it seems reasonable to assume that the lesion is congenital. Smith et al. speculated that the disease arises from a failure of a caliber-persistent artery running within the submucosa to differentiate into capillaries [5]. When the right side is involved, Stoopen’s theory of avaried embryologic development of the right bronchial artery leading to a higher risk for right anomalous vessels points towards a congenital etiology [2]. Most authors favor a congenital origin, although chronic inflammation and smoking may serve as a possible etiological or contributory factors [6, 25, 31, 33].

An exhaustive diagnostic evaluation was performed with chest X-rays, CT scans, bronchoscopies, biopsies, and angiographies in the historical cases. To exclude an underlying lesion potentially responsible for hemoptysis, most patients underwent conventional chest X-rays and CT scans. 28 cases were characterized by chest roentgenograms; however, the results of most were normal (16 cases). 60 patients underwent CT scans, the hallmarks of CT scans were ground glass opacities (24 cases), followed by normal findings (9 cases). Accordingly, chest X-rays and CTs were unable to establish a diagnosis of Dieulafoy’s disease. Nevertheless, CT scans demonstrated no parenchymal abnormalities, except ground glass opacities, reflecting the severity of bleeding [31]. A preliminary inspection was made to exclude other lung diseases causing bleeding.

**Fig. 3:**
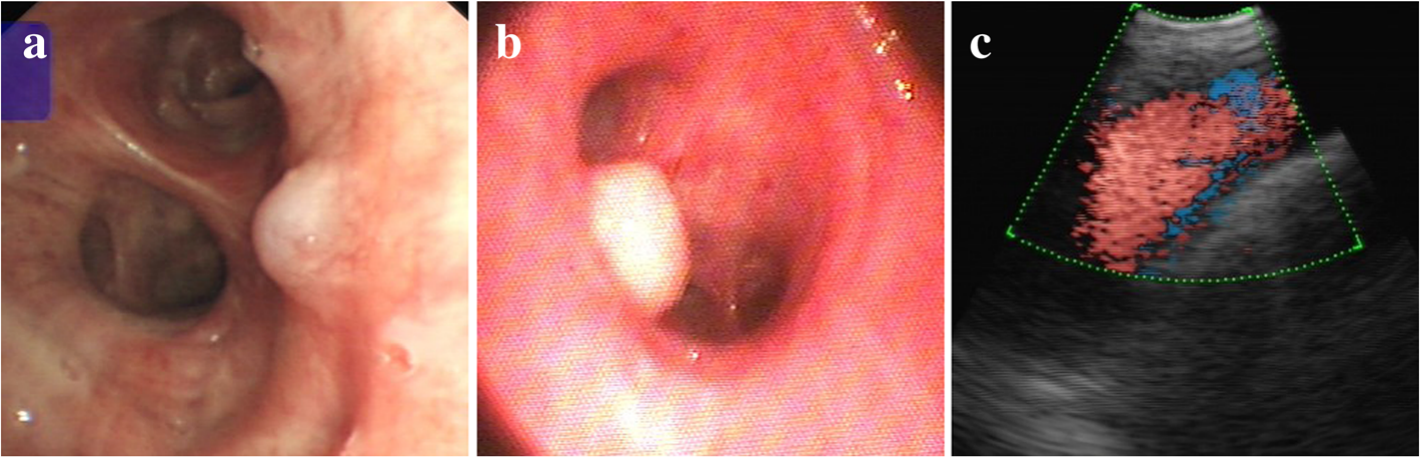
The major manifestation of Dieulafoy’s disease in the bronchoscopy and endobronchial ultrasound. Bronchoscopy showed a nodule with smooth mucosa in the bronchus (**a**, **b**). Endobronchial ultrasound detected a fluid echo-free zone in the submucosal lesion, and the Doppler mode can detect a blood flow (**c**)

Bronchoscopies mainly showed a nodular or protruding lesion with smooth mucosa (39/64) (Fig. 3a and b), only clot or blood without other appearances (15/64), and only bleeding points (5/64). The usual protruding surface is with bronchial mucosa and a white cap, easily misdiagnosed as endobronchial tumor nodule [31] (Fig. 3b). Unlike gastrointestinal diseases in which the endoscopic findings are diagnostic, the bronchoscopic findings in Dieulafoy’s disease is not diagnostic because the site where the abnormal vessel opens into the bronchus is usually a pinpoint mucosal defect surrounded by normal-appearing mucosa and a small lesion (usually < 10 mm) may be difficult to detect due to pooling of blood or filling of the bronchial lumen with clots [6]. Moreover, bronchial biopsies in such diseases entail the risk of triggering sudden massive hemoptysis, but are still frequently attempted (19/57). Biopsies are frequently attempted because the cases mostly appear as a nodular lesion without a typical vascular lesion being easy misdiagnosed for an endobronchial mass or carcinoid tumor, and some patients are admitted to the hospital due to others diseases or symptoms without hemoptysis. However, the biopsy specimens are usually non-diagnostic due to the results of which mainly showed a normal bronchial mucosa with conserved structures, and the biopsy pathological specimens were mostly not ideal because of contamination by bleeding.

Four patients were shown to have vascular malformations under mucosal protrusion by endobronchial ultrasound scan (EBUS) [11, 21, 32, 44]. EBUS bronchoscopes with integrated probes at the distal ends usually have a Doppler mode and can be used to differentiate vascular from solid endobronchial masses, and therefore may help avoid potentially disastrous interventions [47, 48]. Color-Doppler examination performed on the mucosal lesion usually showed a little vessel with arterial flow below the surface (Fig. 3c). Bronchial angiography can show dilated bronchial arteries, abnormal arteries, vascular shunts, fistulae, and extravasation of contrast into the lungs [27]. Lesions of the pulmonary arteries may not be visualized. In such situations, there may be some role for EBUS during bronchoscopy to further evaluate suspicious lesions, or pulmonary angiography may be essential. Thus, if such a lesion is either recognized or suspected, vascular arteriography and EBUS as a diagnostic method should be the preferred choice as the initial investigation rather than chest X-ray, CT scan, and bronchial biopsy.

Management options have included conservative treatment, surgical lung resection, BAE, and bronchoscopic ablation. Conservative treatment is not usually effective because the hemorrhage is almost always from the abnormal vessels. Bronchoscopic ablation has been attempted in a minority of cases and only five cases have been published. The location of the lesion and vascular condition for attempted bronchoscopic intervention are vital, and may determine success or failure. Sheth et al. [38] expressed concern about the location of the lesion on the posterior membrane with esophagus running immediately adjacent posteriorly. Mediastinitis, esophageal injuries, and bronchoesophageal fistulas are all potential complications. Moreover, when the integrity of the abnormal vessel is lost with rapid, abundant bleeding, the field of view is not clear, which is unfavorable for an ablation attempt. According to an analysis of 64 historical cases, only 7.8% of the cases showed bleeding point. Thus, bronchoscopic ablation is an alternative treatment, but the effect has yet to be discussed.

Selective therapeutic BAE is often performed as the initial management for hemoptysis, including massive episodes [27, 28], because BAE is less invasive than surgery and both physicians and patients prefer BAE as the first attempt to stop the bleeding [9]. BAE may not always be feasible and the failure rate of BAE is not negligible. According to this study, 52.6% patients who underwent first-line BAE required lobectomy due to BAE failure or a subsequent recurrence. The poor efficacy of BAE in this situation has been attributed to revascularization and neoangiogenesis at the site of therapeutic embolization or hypertrophy of nearby submucosal vasculature creating recurrences after successful procedures [28, 38], broncho-pulmonary shunts [11], and an aberrant pulmonary artery instead of a bronchial artery [2]. Patients with Dieulafoy’s disease treated with embolotherapy may develop a recurrence, hence should be under regular follow-up care [28]. Surgery has been the main definitive treatment for BDD because the bleeding in these cases is massive, often recurrent, and potentially fatal, and this therapy had a success rate of nearly 100% in all reports [6]. While most reports indicated that there were no postoperative recurrences of hemoptysis, long-term follow-up was not documented.

## Conclusions

BDD should always be included in the differential diagnosis of any patient with massive and recurrent hemoptysis. BDD was mainly located in the right bronchus and the abnormal bronchial or pulmonary arteries are a potential source of hemorrhage. This disease should be considered in heavy smokers and middle-aged patients who have atypical chest imaging findings and present with unexplained hemoptysis, especially if the bleeding recurs and is confined to the right bronchus. Useless and dangerous bronchial biopsies should be avoided. Finally, vascular angiography and EBUS may be undertaken to preliminarily diagnose this condition and BAE may be used in stable patients, or patients who cannot tolerate surgery. While, in uncontrolled cases or following BAE failure, surgical treatment as a lifesaving approach that eliminates the possibility of recurrence is best choice and allows accurate histopathological diagnosis of the disease.

## Additional file

Additional file 1: **Table S1.** Clinical characteristics of Bronchial Dieulafoy’s disease. (DOCX 89 kb)

## Data Availability

All data generated or analysed during this study are included in this published article and its supplementary information files.

## Abbreviations

BAE: Bronchial artery embolization
BDD: Bronchial Dieulafoy disease
CMR: Clinical manifestation rate
CT: Computed tomography
EBUS: Endobronchial ultrasound scan
GGO: Ground glass opacities
