# Supplementary material for: Bronchial Dieulafoy’s disease: a retrospective analysis of 73 cases
Source: BMC Pulm Med. 2019 Jun 6;19:104. doi: 10.1186/s12890-019-0863-1 (PMC6555732; doi:10.1186/s12890-019-0863-1)
Supplement: Supplementary file 1 — Table S1. Clinical characteristics of Bronchial Dieulafoy’s disease. (DOCX 89 kb) [file 12890_2019_863_MOESM1_ESM.docx]

**Additional file 1: Table S1.** Clinical characteristics of Bronchial Dieulafoy’s disease

| Patient | Country | Sex | Age (years) | Smoke  (pack year) | Respiratory history | Reason for admission | Previous bleeding | X-ray | CT | Bronchoscopy | Biopsy and hemoptysis | Angiography | PE | Location | Souce of vessel | Treatment |
| --- | --- | --- | --- | --- | --- | --- | --- | --- | --- | --- | --- | --- | --- | --- | --- | --- |
| Sweerts et al 1995[[3](#_ENREF_3)] | UK | F | 35 | 0 | NO | Massive hemoptysis | YES | Normal | Normal | Clot in RLL  bronchus and  pulsating bleeding point. | NO Biopsy | Normal | NA | RLL | BA | BAE (failed)+  Surgery |
| Sweerts et al 1995[[3](#_ENREF_3)] | UK | M | 59 | YES(40 cigarettes  /day) | NA | Hemoptysis | NO | NA | N/A | RLL bronchus  filled with blood | NA | NA | NA | RLL | BA | Surgery |
| Hiroshi Soda et al 1995[[17](#_ENREF_17)] | Japan | M | 52 | YES(273p/y) | NO | Recurrent hemoptysis | YES | Normal | Normal | Blood clot and an non-pulsatile protrusion produced brisk bleeding in RUL | NO biopsy | Enlarged and convoluted vessels with BA shunts | a prominent artery with an irregularly thickened wall | RUL | BA | Surgery |
| Antoune et al 1998[[47](#_ENREF_47)] | NA | M | 45 | NA | NA | NA | NA | NA | NA | NA | NA | NA | NA | RUL | NA | NA |
| Antoune et al  1998[[47](#_ENREF_47)] | NA | M | 56 | NA | NA | NA | NA | NA | NA | NA | NA | NA | NA | LLL | NA | NA |
| Antoune et al 1998[[47](#_ENREF_47)] | NA | M | 69 | NA | NA | NA | NA | NA | NA | NA | NA | NA | NA | RUL | NA | NA |
| Vander et al 1999[[25](#_ENREF_25)] | Netherlands | F | 70 | YES  (60p/y) | TB, COPD | Acute lobar pneumonia | YES | Normal | NA | 8mm lesion in  LUL covered  with normal  mucosa | Massive bleeding after biopsy | NA | BS indeed contained vascular wall | LUL+RLL | PA | Dead |
| Stoopen et al 2001[[2](#_ENREF_2)] | Mexico | M | 51 | YES(40 p/y) | NO | Massive hemoptysis | YES | A diffuse opacity | An intraluminal mass | Active arterial bleeding point at RML bronchus | None | NA | NA | RML | NA | contact electrocautery （failed）+Surgery |
| Maxeiner et al 2001[[26](#_ENREF_26)] | Germany | M | 62 | NA | Bronchiectasisbronchitis pneumonias | Bronchiectasis and bronchitis | NO | N/A | spotted and striated shadows | A endobronchial protrusion with normal mucosa in RML | Massive bleeding after biopsy,  then dead | NA | BS included chronic bronchitis, scarring, and no malignancy  Autopsy：a tortuous bronchial artery of abnormal | RML+RLL | BA | Dead |
| Hope-Gill et al 2002[[27](#_ENREF_27)] | UK | M | 49 | YES(30 p/y) | TB | Massive hemoptysis | YES | Several small, tissue-dense opacities | NA | Non pulsatile  lesion in the posterior segment of the RUL | NO biopsy | a RBA arising from the third right intercostal artery supplying hypervascular area | NA | RUL | BA | BAE(sucess) |
| Bhatia et al 2003[[28](#_ENREF_28)] | UK | M | 42 | YES | TB | Massive hemoptysis | YES | Bilateral upper lobe opacities | Apical pleural thickening and numerous cavities | Normal | NO biopsy | The RBA was dilated and irregular | NA | RUL | BA | Recurrent BAE |
| Kuzucu et al 2005[[6](#_ENREF_6)] | Turkey | M | 28 | YES(15 p/y) | TB | Massive hemoptysis | YES | Normal | a nodular lesion | A nodular lesion in LLL | Little bleeding after Biopsy | NA | NA | LLL | NA | Surgery |
| Kuzucu et al 2005[[6](#_ENREF_6)] | Turkey | M | 45 | YES(35 p/y) | NO | Massive hemoptysis | YES | Peribronchial  lymphadenopathy | peribronchial  lymphadenopathy | The bleeding source in RML | Biopsy, but no bleeding | a tortuous, dilated RBA originating from the aorta at the level of the sixth thoracic vertebra | BS were nondiagnostic | RML | BA | BAE (failed)+  Surgery |
| Pomplun et al 2005[[29](#_ENREF_29)] | UK | M | 32 | NA | NO | Massive hemoptysis | NO | NA | A enhancing  mass lesion | NA | No biopsy | NA | a large PA opened into the bronchial lumen | RUL | PA | BAE (failed)+  Surgery |
| Löschhorn et al  2006  [[30](#_ENREF_30)] | Switzerland | F | 47 | NA | NO | Memoptysis | NO | NA | NA | Lesion is 5mm in diameter and 1 mm high in RMB + sessile tumor in the intermediate  bronchus | Massive bleeding after biopsy | a tortuous, dilated bronchial artery | a tortuous BA branch hug the bronchus, reaching to the mucosal surface | RMB+ right intermediate bronchus | BA | Surgery |
| Löschhorn et al 2006  [[30](#_ENREF_30)] | Switzerland | F | 52 | NA | NO | Hemoptysis | NO | Normal | GGO | Two 3-5mm  lesions at the RLL covered by mucosa. both had a white, pointed cap | No biopsy | a hypertrophic right bronchial artery and an abnormal arterial plexus in RLL | none | RLL | BA | BAE |
| Xie et al  2006 n°16  [[14](#_ENREF_14)] | China | F | 28 | No | NO | Massive hemoptysis | NO | An opacity in LLL | Flaky opacity | The nodule with normal mucosa, had a white, pointed cap | Biopsy,  but no bleeding | NA | BS: inflammatory exudate | LLL | PA | Conservative treatment |
| Rennert et el  2007  [[42](#_ENREF_42)]  Gharagozloo 2008[[44](#_ENREF_44)] | USA | M | 51 | YES | Pneumonia | Hemoptysis | YES | Normal | normal | A small sessile mass | Massive bleeding after biopsy | none | BS:NA  large vascular malformation(12–13mm) | LLL | NA | Surgery |
| Fields et el  2008  [[43](#_ENREF_43)] | USA | M | 47 | YES(25 p/y) | NO | Fevers, chills, and cough, hemoptysis | YES | Normal | NA | 22-mm mucosal lesion with intermittent pulsatile bleeding | NA | NA | NA | left main bronchus | NA | Cryotherapy(failed)+ place a  Dumon silicone stent |
| Parrot et al2008  [[31](#_ENREF_31)] | France | M | 69 | YES(60 p/y) | COPD | Massive hemoptysis | YES | Normal | GGO | Bilateral bronchial  flooding by blood was evidenced | NA | NA | NA | RUL | BA | BAE (failed)+  Surgery |
| Parrot et al 2008  [[31](#_ENREF_31)] | France | M | 45 | YES(60 p/y) | NO | Massive hemoptysis | YES | Normal | GGO | Bilateral bronchial  flooding by blood was evidenced | NA | NA | NA | RUL | BA | BAE (successful)+  Surgery |
| Parrot et al 2008  [[31](#_ENREF_31)] | France | M | 54 | YES(30 p/y) | Chronic bronchitis | Massive hemoptysis | NO | Normal | GGO | Bilateral bronchial  flooding by blood was evidenced | NA | NA | NA | LLL | BA | BAE (failed)+  Surgery |
| Parrot et al 2008[[31](#_ENREF_31)] | France | F | 57 | YES(50 p/y) | TB | Massive hemoptysis | YES | Normal | GGO | Bilateral bronchial  flooding by blood was evidenced | NA | NA | NA | LUL | BA | BAE (failed)+  Surgery |
| Parrot et al 2008  [[31](#_ENREF_31)] | France | M | 49 | YES(30 p/y) | NO | Massive hemoptysis | NO | Normal | GGO | Bilateral bronchial  flooding by blood was evidenced | NA | NA | NA | LUL | BA | BAE (failed)+  Surgery |
| Parrot et al 2008  [[31](#_ENREF_31)] | France | M | 38 | YES(13 p/y) | NO | Massive hemoptysis | YES | Normal | GGO | Bilateral bronchial  flooding by blood was evidenced | NA | NA | NA | RLL | BA | BAE (failed)+  Surgery |
| Parrot et al 2008  [[31](#_ENREF_31)] | France | F | 68 | YES(100 p/y) | NO | Massive hemoptysis | YES | Normal | GGO | Bilateral bronchial  flooding by blood was evidenced | NA | NA | NA | RML | BA | Surgery |
| Gurioli. C et al 2010  [[32](#_ENREF_32)] | Italy | M | 65 | YES | Asthma | Dyspnea | NO | NA | Bilateral micronodules | A small protrusion with a white cap in right intermediate  bronchus | bleeding after Biopsy | NA | BS: a part of a wall of a small sized arterial vessel | Right intermediate  bronchus | NA | Conservative treatment |
| D’Souza et al 2010  [[46](#_ENREF_46)] | Australia | F | 63 | NA | Bronchiectasis and chronic bronchitis | Bronchiectasis and bronchitis | No | A mass lesion | Bronchiectatic changes only | 3mm protruding  lesion in RMB | Persistent haemorrhage after Biopsy | NA | Autopsy: a blood vessel within the submucosa extending towards the luminal surface | RMB | BA | Dead |
| WAN et al 2011[[18](#_ENREF_18)] | China | M | 19 | NO | NO | Recurrent hemoptysis | YES | NA | Inflammatory | bleeding clots; nodular, mucosal lesions in RML | bleeding after biopsy | Abnormal communication between the diaphragm artery and the BA | BS: vascular malformation | RML | BA | BAE(failed)+  Surgery |
| Barisione et al 2012  [[4](#_ENREF_4)] | Italy | F | 57 | No | NO | Massive hemoptysis | YES | NA | Normal | a 1–2 mm lesion with normal mucosa in RML | massive  hemoptysis after biopsy | convoluted and ectatic bronchial vascular structures | BS：normal bronchial mucosa with conserved structure | RML | BA | BAE |
| Kolb et al 2012  [[40](#_ENREF_40)] | USA | F | 44 | NA | NO | Hemoptysis. | YES | NA | NA | A tortuous, nonpulsatile  vessel beneath the bronchial mucosa | NO biopsy | hypertrophied tortuous bronchial artery arising from the distal aortic arch, anastomosis between BA and PA | NA | RML | BA | BAE |
| Trisolini et al 2013[[36](#_ENREF_36)] | Italy | F | 66 | NA | bronchiectasis | recurrent pneumonias in LLL | NO | NA | NA | two nodular, nonpulsating mucosal lesions in LLL | massive bleeding after biopsy | NA | tortuous, large BA frequently running in the bronchial submucosa | LLL | BA | Surgery |
| Yang et al 2013  [[13](#_ENREF_13)] | China | M | 41 | No | NO | Massive Hemoptysis | YES | GGO | GGO | a white cap | NO biopsy | NA | Abnormal blood vessels form hemangioma-like structures in the bronchial wall | RML | NA | Surgery |
| Yang et al 2013  [[13](#_ENREF_13)] | China | M | 36 | NA | Pneumonia | Pneumonia | No | NA | Inflammatory manifestations | A tortuous, nonpulsatile  lesion beneath the bronchial mucosa | massive  hemoptysis after biopsy, then dead | NA | Autopsy :part of a wall of pumonaray vessel | Right intermediate  bronchus | PA | Dead |
| Yang et al 2013  [[13](#_ENREF_13)] | China | M | 61 | NA | NA | Massive hemoptysis | YES | NA | GGO | Bleeding in RUL | NO biopsy | NA | torturous artery extending into the  bronchial mucosa | RUL | NA | Surgery |
| Smith et al 2014  [[5](#_ENREF_5)] | Australia | M | 30 | No | NO | Massive hemoptysis | YES | NA | GGO | fresh bleeding from the basal  segments of the RLL | NO biopsy | Normal | a large torturous artery extending into the bronchial mucosa | RLL | NA | BAE (failed)+  Sur gery |
| Fang et al 2014  [[7](#_ENREF_7)] Ganganah et al  2015  [[11](#_ENREF_11)] | China | M | 13 | No | NO | Massive hemoptysis | No | NA | GGO | two non-pulsating nodules in 1.5 mm high with white caps covering normal mucosa in RLL | NO biopsy | a dilated and tortuous RBA arising from the thoracic aorta | Small vessels and a tortuous BA opened directly into bronchial lumens | RLL | BA | BAE (failed)+  Surgery |
| LIU et al 2014[[19](#_ENREF_19)] | China | F | 18 | NO | NO | Recurrent hemoptysis | YES | NA | fibrous cord, Atelectasis in RLL | Submucosal purple blood vessel distribution | NO biopsy | Tortuous branches of the bronchial artery | Abnormal vessels | RLL | BA | Surgery |
| LIU et al 2014[[19](#_ENREF_19)] | China | M | 23 | YES(100p/y) | NO | Hemoptysis | NO | NA | Flaky shadows | a small uplifted nodule with a smooth surface | NO biopsy | Dilated branches of the bronchial artery | NO | LUL | BA | Conservative treatment |
| LIU et al 2014[[19](#_ENREF_19)] | China | M | 31 | YES(200p/y) | surgery for pulmonary bullae | Hemoptysis | NO | NA | Flaky oozing shadow | Two uplifted nodules with a smooth surface | NO biopsy | Tortuous branches of the bronchial artery | NO | RLL | BA | BAE |
| LIU et al 2014[[19](#_ENREF_19)] | China | F | 33 | NO | NO | Recurrent hemoptysis | YES | NA | Flaky oozing shadow | a uplifted nodule with a smooth surface | NO biopsy | Bilateral bronchial arteries are distorted, some tumor-like dilatation | NO | RLL | BA | BAE |
| LIU et al 2014[[19](#_ENREF_19)] | China | F | 36 | NO | NO | Hemoptysis and cough | YES | NA | Atelectasis, Small amount of pleural effusion | Two uplifted nodules with a smooth surface | NO biopsy | Tortuous branches of the bronchial artery | NO | RLL | BA | BAE |
| LIU et al 2014[[19](#_ENREF_19)] | China | F | 47 | NO | NO | Right chest pain | NO | NA | Bronchial wall thickening | A nodule with white caps | NO biopsy | the bronchial artery is tortuous, dilated | NO | RML | BA | Conservative treatment |
| Dalar et al  2015  [[12](#_ENREF_12)] | Turkey | M | 28 | YES(1p/y) | NO | Massive hemoptysis | YES | NA | Normal | 3mm sessile  lesion covered by mucosa at the  entrance of LUL | NO Biopsy | NA | none | LUL | NA | argon plasma coagulation |
| Padilla et al  2015  [[33](#_ENREF_33)] | spain | F | 49 | YES(29 p/y) | COPD | Massive hemoptysis | No | NA | Normal | plentiful blood remnants and signs of chronic inflammation | NO Biopsy | a tortuous and dilated RBA and LBA | none | LL+  RL | BA | BAE |
| Xia et al  2015  [[15](#_ENREF_15)] | china | M | 31 | YES(15 p/y) | NA | Hemoptysis | NA | Infiltrate and GGO | Infiltrate and GGO | nodules about  1 mm high above the surface | NA | NA | superficial dysplastic BA under the bronchial epithelium | RML | BA | BAE (failed)+  Surgery |
| Xia et al  2015  [[15](#_ENREF_15)] | china | M | 21 | YES(5 p/y) | NA | Hemoptysis | NA | Infiltrate and GGO | Infiltrate and GGO | nodules about  1 mm high above the surface | NA | NA | superficial dysplastic BA under the bronchial epithelium | RML | BA | BAE (failed)+  Surgery |
| Xia et al  2015  [[15](#_ENREF_15)] | china | M | 85 | YES(40 p/y) | NA | Hemoptysis | NA | Infiltrate and GGO | Infiltrate and GGO | nodules about  1 mm high above the surface | NA | NA | superficial dysplastic BA under the bronchial epithelium | LUL | BA | BAE (failed)+  Surgery |
| Xia et al  2015  [[15](#_ENREF_15)] | china | M | 63 | YES(10 p/y) | NA | Hemoptysis | NA | Infiltrate and GGO | Infiltrate and GGO | nodules about  1 mm high above the surface | NA | NA | superficial dysplastic BA under the bronchial epithelium | LUL | BA | Surgery |
| C. Lin et al 2015 [[45](#_ENREF_45)] | USA | M | 55 | NA | NO | hemoptysis | YES | NA | NA | a clot in his RMB，A sub centimeter sessile lesion covered with mucosa | NO biopsy | a hypertrophied RBA bifurcating into a tortuous plexus of dilated branches | NO | RMB | BA | BAE |
| **Viola** et al  2016  [[34](#_ENREF_34)] | London | F | 83 | No | NO | Hemoptysis | YES | Normal | Normal | Lesion in RUL  bronchus <1cm | Massive  bleeding | NA | NA | RUL | NA | Surgery |
| Venus et al  2016[[20](#_ENREF_20)] | India | M | 45 | NO | NO | hemoptysis | NO | NA | bilateral lower lobe and RML  flaky oozing shadow | the bleeders were identified on smooth mildly elevated mucosa | NO Biopsy | Tortuous branches of the bronchial artery | large and  dysplastic bronchial artery in the submucosal | RLL | BA | BAE(failed)+  Surgery |
| Venus et al  2016[[20](#_ENREF_20)] | India | M | 41 | YES | NO | massive hemoptysis | NO | NA | Fluid levels  within the bronchi in RLL,GGO | the bleeders were identified on smooth mildly elevated mucosa | NO Biopsy | Tortuous branches of the bronchial artery | tortuosity of BA reaching up to the mucosa of the bronchi | LLL | BA | BAE(failed)+  Surgery |
| Venus et al  2016[[20](#_ENREF_20)] | India | M | 78 | YES | NO | hemoptysis | NO | NA | bilateral  rounded alveolar opacities with GGO | the bleeders were identified on smooth mildly elevated mucosa | NO Biopsy | Tortuous branches of the bronchial artery | large and  dysplastic bronchial artery in the submucosa | LUL | BA | BAE(failed)+  Surgery |
| Niu et al  2017  [[16](#_ENREF_16)] | china | F | 8 month | No | NO | Hemoptysis | YES | NA | Flaky blur | Bleeding in RUL | NO | The blood supply of RUL is abundant, and the bronchial arteries are dilated | Malformed thin-walled blood vessels rupture on the mucosal surface | RUL | BA | BAE (failed)+  Surgery |
| Hadjiphilippou et al  2017  [[35](#_ENREF_35)] | UK | M | 47 | YES(70 p/y) | NO | Hemoptysis | YES | NA | NA | a small polypoid lesion in RLL | NO Biopsy | two large BA were extending from the thoracic aorta to the right hilum and RUL | none | RLL | BA | BAE |
| Madan et al 2017  [[10](#_ENREF_10)] | India | M | 26 | No | NO | Recurrent hemoptysis | YES | NA | GGO and focal bronchiectasis | Extensive blood clots ,and active mucosal blood spurting in RLL, mucosal vascular tortuosity was visible | NO Biopsy | NA | none | RLL | NA | argon plasma coagulation |
| Wadji et al  2017  [[9](#_ENREF_9)] | Iran | F | 16 | No | NO | Massive hemoptysis and respiratory failure | YES | Normal | A very small lesion | a lesion with normal mucosa in RLL | NO Biopsy | NA | few dilated vessels in the submucosa | RLL | NA | Surgery |
| Yang et al 2017  [[8](#_ENREF_8)] | China | F | 60 | No | NA | Frequent hemoptysis | YES | NA | Normal | a 1-cm lesion near the carina. Tortuous blood vessels were observed | NO Biopsy | an artery extending into the submucosa from the descending aorta | none | Near carina | NA | BAE |
| Lentz et al 2016  [[39](#_ENREF_39)]  Sheth et al 2018  [[38](#_ENREF_38)] | USA | F | 51 | NA | Branch pulmonary artery stenoses | Massive hemoptysis | No | NA | NA | A 5mm nodular lesion in LLL, mucosal vascular tortuosity was visible | NO Biopsy | NA | none | LLL | NA | Nd：YAP激光 |
| Sheth et al  2018  [[38](#_ENREF_38)] | USA | M | 76 | YES | NO | Hemoptysis | No | NA | a hypertrophied  bronchial artery supplying | Clotted blood in both mainstem bronchi, A 1 to 2mm nodular lesion in RML, A 3mm nodular lesion in LML | NO Biopsy | several additional branches of the same  hypertrophied artery seeming to supply the region | none | RML  +LML | BA | BAE |
| Bonnefoy et al  2018[[37](#_ENREF_37)] | France | M | 66 | YES | COPD | Massive hemoptysis | NA | NA | Normal | persistent bloody | NO Biopsy | NO | superficial dysplastic artery located into the submucosa close to bronchial lumen | RUL | BA | BAE(failed)+  Surgery |
| Pan et al  2018[[21](#_ENREF_21)] | China | F | 76 | NO | has a history of tuberculosis | Cough | NO | NO | Pulmonary interstitial thickening | nodular lesion in RML+ RLL | Massive  bleeding | a tortuous, dilated bronchial artery | Abnormal vascular structure | RML+ RUL | BA | Conservative treatment |
| Pan et al  2018[[21](#_ENREF_21)] | China | M | 66 | YES(25p/y) | NO | Hemoptysis | NA | NO | Bronchiectatic changes and Inflammatory, Atelectasis | pulsating vessel in RML, nodules about 1 mm high above the surface in LLL | NO Biopsy | a tortuous, dilated bronchial artery | NO | RUL+RML+RLL+LUL | BA | Conservative treatment |
| Pan et al  2018[[21](#_ENREF_21)] | China | M | 51 | YES(60p/y) | NO | Cough and fever | NO | NO | Left upper lobe cavity, fibrous cord, Atelectasis | Normal | NO Biopsy | a tortuous, dilated bronchial artery | NO | RUL+RML | BA | BAE |
| Pan et al  2018[[21](#_ENREF_21)] | China | M | 36 | NO | NO | Recurrent pulmonary infections | YES | NO | spotted shadows | NA | Massive  bleeding | NO | NO | RLL | NA | Dead |
| Pan et al  2018[[21](#_ENREF_21)] | China | M | 61 | NO | NO | Hemoptysis | NA | NO | GGO | NA | NO Biopsy | NO | Abnormal vessels | NA | NA | Surgery |
| Pan et al  2018[[21](#_ENREF_21)] | China | M | 41 | YES(10 p/y) | NO | Recurrent hemoptysis | YES | NO | GGO | NA | NO Biopsy | NO | Abnormal and dilated vessels | RML+RLL | NA | Surgery |
| Wang et al  2018[[22](#_ENREF_22)] | China | F | 21 | NO | NO | Massive hemoptysis | YES | NA | GGO  due to aspiration of the blood in RLL | Bleeding, two apophyses of about 3–5 mm in diameter in RLL | NO Biopsy | Tortuous branches of the bronchial artery, convoluted vessels originated from the thoracic aortic | NO | RLL | BA | BAE |
| Chen et al 2019[[23](#_ENREF_23)] | China | F | 18 | NO | NO | Frequent hemoptysis | YES | NA | GGO in RLL | a nodule at the entrance to RB10 | Bleeding after biopsy | a tortuous right bronchial artery | The pathological specimen is not ideal | RLL | BA | BAE |
| Chen et al 2019[[23](#_ENREF_23)] | China | F | 72 | NA | upper respiratory tract infections | cough and  blood expectoration | YES | NO | RML atelectasis and RLL  bronchiectasis with infection | a small uplifted mucosal lesion with a smooth surface without pulsation in RLL | massive bleeding during the biopsy | NA | NA | RLL | NA | Dead |
| Chen et al 2019[[23](#_ENREF_23)] | China | M | 38 | NO | LBA malformation | Recurrent hemoptysis | YES | left lobe opacities suggestive of pneumonia | GGO, the abnormal LBA, uneven in thickness | No bronchoscopy | NO Biopsy | NA | NO | Left lung | BA | BAE |
| Zhou  Et al  2019[[24](#_ENREF_24)] | China | M | 62 | YES(360p/y | COPD, pulmonary bullae, pneumonia, bronchiectasis | Intermittent hemoptysis | YES | NA | local atelectasisemphysema, pulmonary bullae | a slit-like stenosis ,swollen and smooth mucosa, a significantly wider subsection carina | massive bleeding after biopsy | abundant distortedand hyperplastic bronchial arteries in the LLL | Autopsy: confirmed Dieulafoy's disease of the bronchus | LLL | BA | BAE |

M: male; F: female; GGO: Ground Glass Opacity; LLL: left lower lobe; LUL: left upper lobe; RLL: right lower lobe; RML: right middle lobe; RUL: right upper lobe; RMB: right main bronchus; RBA: right bronchial artery; LBA: left bronchial artery; PE: pathological examination; PA: pulmonary Artery; BA: bronchial artery; BAE: bronchial artery embolization; BS: Biopsy specimens; NA: not available;
